# The Cards and Lottery Task: Validation of a New Paradigm Assessing Decision Making Under Risk in Individuals With Severe Obesity

**DOI:** 10.3389/fpsyt.2020.00690

**Published:** 2020-07-16

**Authors:** Lisa Schäfer, Ricarda Schmidt, Silke M. Müller, Arne Dietrich, Anja Hilbert

**Affiliations:** ^1^Integrated Research and Treatment Center AdiposityDiseases, Research Unit Behavioral Medicine, Department of Psychosomatic Medicine and Psychotherapy, Leipzig University Medical Center, Leipzig, Germany; ^2^Department of General Psychology: Cognition and Center for Behavioral Addiction Research, University of Duisburg-Essen, Duisburg, Germany; ^3^Integrated Research and Treatment Center AdiposityDiseases, Department of Visceral, Transplantation, Thoracic and Vascular Surgery, Leipzig University Medical Center, Leipzig, Germany

**Keywords:** obesity, risk taking, decision making, impulsivity, bariatric surgery, neuropsychological task

## Abstract

**Background:**

A growing body of research demonstrated impaired executive functions in individuals with severe obesity, including increased sensitivity to reward and impulsive decision making under risk conditions. For the assessment of decision making in patients with severe obesity, studies widely used the Iowa Gambling Task (IGT) or the Delay Discounting Task (DDT), which cover short-term or long-term consequences of decisions only. A further development originating from the field of addiction research is the Cards and Lottery Task (CLT), in which each decision made has conflicting immediate and long-term consequences at the same time. The present study aimed to validate the CLT in individuals with severe obesity.

**Methods:**

Patients with severe obesity *(N* = 78, 67% women, 42.9 ± 10.4 years old, body mass index of 48.1 ± 8.3 kg/m^2^) were included. Convergent validity was evaluated using the computerized Delay Discounting Task and well-established self-report questionnaires assessing different aspects of impulsivity. For discriminant validity, CLT performance was compared between symptom groups characterized by high *versus* low impulsivity. The task’s clinical validity was evaluated based on associations with general and eating disorder psychopathology, and body mass index. Test-retest reliability was determined by administering the CLT in *n* = 31 participants without weight-loss treatment one year later. The task’s sensitivity to change due to weight loss was evaluated by retesting *n* = 32 patients one year after receiving obesity surgery.

**Results:**

The number of advantageous decisions in the CLT was significantly positively associated with delay discounting and effortful control, and significantly negatively correlated with behavioral impulsivity. CLT performance differed significantly between individuals with and without symptoms of attention-deficit/hyperactivity disorder and between samples with severe obesity and healthy controls. Clinically, CLT performance was significantly associated with general, but not eating disorder psychopathology. The CLT showed moderate test-retest reliability after one year in weight-stable individuals and was sensitive to change in those undergoing obesity surgery.

**Conclusions:**

This study identified the CLT to be a highly promising, new complex measure of short- and long-term decision making with good reliability and validity in individuals with severe obesity. Future studies should assess its association with the IGT and predictive value for real-life health behavior.

## Introduction

The worldwide prevalence of obesity, defined as a body mass index (BMI, kg/m²) ≥ 30 kg/m², is approaching pandemic extent (1). Particularly the prevalence of severe obesity (grade 3, BMI ≥40 kg/m²) is continuously increasing (2), thereby escalating physical and mental comorbidities, such as hypertension, type 2 diabetes mellitus, and dyslipidemia, as well as affective disorders (3, 4). Although behavioral weight-loss treatment, subsuming dietary, physical, and behavioral interventions, is the standard intervention for obesity (5), its effects are moderate, with a mean weight loss of 2.4 kg over 12–18 months (6). In contrast, surgical weight-loss interventions produce substantial weight loss (7), although long-term effects (>5 years) are variable across patients and weight regain is likely to set in again (8, 9), particularly in those with a pre-surgery BMI ≥ 40 kg/m² (10). Understanding the precise etiological mechanisms that promote weight gain and prevent successful weight-loss maintenance is essential to develop effective interventions in the prevention and treatment of obesity.

During the past decade, neuropsychological research identified executive functions to play a crucial role for the development of obesity and weight-loss success [e.g., (11, 12)]. Executive functions describe higher cognitive processes that enable to manage impulses and goal-directed behavior (13, 14) including inhibition, cognitive flexibility, working memory, planning, and decision making (15, 16). A recent meta-analysis including 72 experimental studies revealed significant impairments in executive functions in individuals with obesity compared to those with normal weight, with largest effects in decision making [Hedges g = −0.44, (17)], predominantly assessed by the Iowa Gambling Task [IGT (18)] and the Delay Discounting Task [DDT (19)]. Participants’ BMI did not moderate the effects (17); however, most evidence based on a narrow BMI across the obesity range. Indeed, only a minority of studies on executive functions, specifically decision making, was conducted in samples with severe obesity. The few available studies examining samples with a mean BMI ≥ 40 kg/m² consistently revealed deficient decision making compared to controls with normal weight based on the IGT (20–23), but not based on the DDT (19, 24). Although there is growing evidence on improved attention, memory, and cognitive flexibility after behavioral and surgical weight-loss interventions (25), longitudinal data on the effects of weight loss on decision making is sparse. In *N* = 16 patients undergoing gastric bypass, IGT scores did not significantly change from pre- to 24 weeks post-surgery (26). Nevertheless, the implications of these findings and their transferability to everyday-life decision making are still not clear, especially due to the methodological aspects of the instruments used.

The IGT is a popular neuropsychological measure which was designed to assess decision making in a learning context (18). During the IGT, participants are shown four hidden decks of cards and instructed to choose one of these decks with the ultimate goal to maximize virtual money. Each card deck has a certain probability of gaining and losing virtual money, which is thought to be learned by the participant during the 100 trials. In the long-run, two of the decks are more disadvantageous than the other two decks, as they lead to more losses. Because the rules for gains and losses are not explicitly presented to the participant, the IGT, particularly at the beginning of the task, is a measure of decision making under ambiguous, rather than objective risk conditions.

Importantly, in addition to the amount and frequency of rewards which are considered by the IGT, everyday-life decision making involves choices that differ in their timing of consequences. Temporal discounting, which is assessed during DDTs, is a specific element of decision making describing the depreciation of the value of a reward (e.g., money or food) related to the time that it takes to be released. For example, individuals might choose 100 EUR delivered immediately over 200 EUR to be delivered in two years. Specifically, DDTs determine an indifference point at which participants forgo delayed rewards in favor of immediate rewards, although the delayed rewards are objectively more valuable. Although delay discounting involves a dual-process competition [reflective *versus* impulsive system, (27)], the decision is only accompanied by either a long-term or a short-term consequence, but it does not involve the integration of both at the same time. Due to the longer time interval for receiving the long-term reward (up to years) compared to the IGT, decision processes might be biased by individual’s internal risk evaluations for actually obtaining the reward, making risk conditions ambiguous rather than objective.

The most commonly used reward in the IGT and DDT is money (28), as it represents a “common currency” among individuals of the same culture. Although hypothetically played for, participants were found to discount fictitious amounts of money as steeply as real money (29). Importantly, previous research on the DDT showed high correlations between tasks using money and other types of reward, such as food (30, 31), including samples with obesity (28). The fact that the degree of discounting for one type of reward was consistently positively associated with the degree of discounting for other types of reward including money (32) indicates that delay discounting and impulsive decision making are relatively stable across different decision modalities. This is an important aspect in transferring laboratory-based decision making into real-life settings, where decision making involves complex balancing processes integrating competing short- and long-term consequences at the same time. For example, establishing a healthy lifestyle in the long-term goes along with experiencing short-term punishment, such as exertion from physical activity, or sustaining from preferred, high-caloric foods. Individuals who are sensitive to punishment (i.e., cannot stand negative short-term consequences) are likely to fail maintaining their long-term objective. Extant tasks on decision making as the IGT or Game of Dice Task, however, are designed in a way that those high in punishment sensitivity decide to draw from the save stack without frequent losses, and thus will perform better in the end [see **Table 1**, (33, 34)]. In this context, there is a need to rethink decision-making paradigms and to increase their ecological validity (35–38).

**Table 1:** Comparison of commonly used tasks assessing decision making (Iowa Gambling Task, Delay Discounting Task), and with the newly developed Cards and Lottery Task.

	Iowa Gambling Task	Delay Discounting Task	Cards and Lottery Task
**Rule learning**	**Yes**	**No**	**No**
**Risk conditions**	**Ambiguous** (at least in the beginning)	**Explicit** (immediate reward) **Ambiguous** (choosing a longer time delay for rewards carries the risk of unexpected incidents that prevent the rewards from being paid)	**Explicit**
**Reward sensitivity/Delay of gratification**	High reward sensitivity (low delay of gratification) leads to neglecting negative long-term outcomes in favor of immediate gains and, therefore, to more frequent drawing from deck A, B **high reward sensitivity = worse performance in the IGT**	Not applicable	High reward sensitivity (low delay of gratification) leads to neglecting negative long-term outcomes in favor of immediate gains and, therefore, to more frequent drawing from the left deck **high reward sensitivity = worse performance in the CLT**
**Punishment sensitivity**	High punishment sensitivity leads to not tolerate frequently money losses and, therefore, to more frequent drawing from deck C, D **high punishment sensitivity = better performance in the IGT**	Not applicable	High punishment sensitivity leads to not tolerate frequently money losses and, therefore, to more frequent drawing from the left deck **high punishment sensitivity = worse performance in the CLT**
**Consequence of each decision/trial**	**Only short-term** consequence (immediate win/loss of money)	**Either short or long-term** consequence	**Competing short-term** (immediate win/loss of money) **and long-term consequences** (star/bomb symbols influencing probability for the subsequent lottery) **at the same time**
**Long-term outcome (overall outcome at the end of the task)**	**Cumulated short-term** outcomes	Not applicable	**Cumulated short-term** outcomes **and long-term stack** (i.e., **lottery win/loss)**
**Time interval for long-term outcome**	At the end of the task, i.e., **within 20 min**. (final account balance of virtual money)	Depending on the decision, either **immediately or 2, 30, 180, or 365 days later**	At the end of the task, i.e., **within 20 min**. (final account balance of virtual money)
**Best strategy**	“**Rigid** decision making”: **Learning to choose exclusively** the decks with low, but more frequent immediate wins (e.g., C, D) than decks with high, but rare immediate wins and higher risk for penalty (e.g., A, B)	Not applicable	“**Flexible** decision making”: Excessive restraint from the short-term deck (e.g., left deck) is not the best strategy: For reaching the highest possible overall outcome, participants have to win the lottery (i.e., **frequently choose the right deck), but not exclusively**
**Explicit information given in each trial**	Current balance of virtual money	Not applicable	Win/loss margin and star/bomb-symbol frequencies in both decks, current balance of virtual money, current lottery stack, current trial number

Bold text indicates essential differential task characteristics.

Recently, Müller and colleagues designed a new decision-making paradigm, the Cards and Lottery Task [CLT, (39)]. During the computerized gambling task, the participant chooses between two card decks with explicit information about the probability of gaining or losing virtual money (short-term consequence) and winning an additional lottery jackpot at the end of the game (long-term consequence). The decks are designed in a way that choosing a card from the one deck leads to immediate gains, while increasing the risk of losing the lottery jackpot at the same time. In contrast, choosing the other deck predominantly leads to immediate losses but an increased chance of winning the lottery jackpot. Thus, each decision has simultaneously short- and long-term outcomes compared to the IGT and DDT, where either short- or long-term consequences follow. The best strategy for maximizing the overall outcome is to update and integrate current information on risk probabilities, involving flexibly switching between options (i.e., card decks) and tolerating short-term punishment (i.e., money losses) for a greater reward at the end (i.e., lottery jackpot). This stands in contrast to the best strategy in IGT, which requires rigidly choosing the learned “good” deck, automatically preferred by individuals with high punishment sensitivity. **Table 1** summarizes the main task characteristics of the IGT, DDT, and CLT.

Within a sample of healthy individuals (*N* = 70), Müller et al. (39) showed that decision-making performance varied highly, but, on average, healthy participants preferred advantageous (mostly long-term) over disadvantageous (mostly short-term) decisions. Contrasting extant evidence in risky decision making mostly demonstrating a lack of association with age and sex [e.g., (17, 37, 40)], men and younger individuals made significantly more advantageous decisions than women and older individuals in the study by Müller et al. (39), albeit the effects were small- to medium-sized. Although education may impact decision making with well-educated individuals outperforming those with lower education in the IGT [e.g., (41, 42)], nothing is known about how education relates to the number of advantageous decisions during the CLT so far. In healthy participants, CLT performance was positively associated with working memory, especially updating abilities and logical thinking. The number of advantageous decisions in the CLT was unrelated to the amount of money earned in the Game of Dice Task [GDT, (43)], assessing short-term decision making under explicit risk, highlighting the conceptual difference between short-term only and conflicting short- and long-term decision making. Unexpectedly, the CLT did not significantly correlate with the DDT, which may be due to methodological limitations of the DDT version used in the Müller et al. study (i.e., unrealistically large amounts of money were offered). Lacking correlations with a Card Sorting Task validated that the CLT does not include any learning component (39). Due to the lack of data on the CLT’s reliability, it is unclear so far whether performance on the CLT is relatively stable over time, such as the DDT (44), or biased by learning effects, as with the IGT (38).

Due to the high relevance of decision making for health behaviors in severe obesity, this study sought to examine the psychometric properties of the newly developed CLT for its use in individuals with severe obesity. As the CLT is the only available task measuring decision making based on conflicting short- and long-term prospects at the same time, but has only been validated in a non-clinical sample of healthy adults, the present study will provide valuable information about the task’s suitability in severe obesity as a more complex task on decision making than extant tasks. Beyond evaluating the sociodemographic correlates of CLT performance in severe obesity including age, sex, and education, the task’s validity and reliability were examined. Specifically, it was hypothesized that the number of advantageous decisions of the CLT 1) will positively correlate with DDT performance and self-report measures of self-control, and will be negatively linked to self-reported reward and punishment sensitivity (convergent validity), 2) will discriminate between clinical symptom groups involving self-regulatory difficulties as well as between individuals with severe obesity versus healthy controls from the population [(39), discriminant validity], 3) will be negatively linked to general and eating disorder psychopathology and weight status (clinical validity), 4) will be stable over 12 months (test-retest reliability), and 5) will improve after significant weight loss (sensitivity to change).

## Materials and Methods

### Participants

Data for the present study were obtained between December 2015 and January 2019 in frame of an eye-tracking experiment investigating longitudinal effects of obesity surgery on attentional processing of visual food cues (Schäfer et al., in revision). Inclusion criteria were age ≥ 18 years and sufficient German language skills. Exclusion criteria were current severe physical or mental disorders (e.g., suicidal tendency, acute psychosis) and uncorrected vision impairment.

The total sample consisted of 78 individuals (66.7% female) with a mean age of 42.9 years (*SD* = 10.4, range 24–69) and a mean BMI of 48.1 kg/m^2^ (*SD* = 8.3, range 33.8–78.9). Among them, 39 participants were patients scheduled for obesity surgery and recruited from the Leipzig University Medical Center during their preparatory visit for the surgery and the Psychosocial Registry for Bariatric Surgery [PRAC, (45)]. The remaining 39 participants were individuals with severe obesity and no ongoing or planned intensive weight-loss treatment. They were matched to the prebariatric patients by age, sex, and BMI, and were recruited from the same clinical institution and the population (e.g., Internet advertisements). Written informed consent was obtained from all participants prior to study participation according to procedures approved by the local Ethics Committee of the University of Leipzig. All participants received a financial compensation for their participation.

### The Cards and Lottery Task

**Figure 1:**
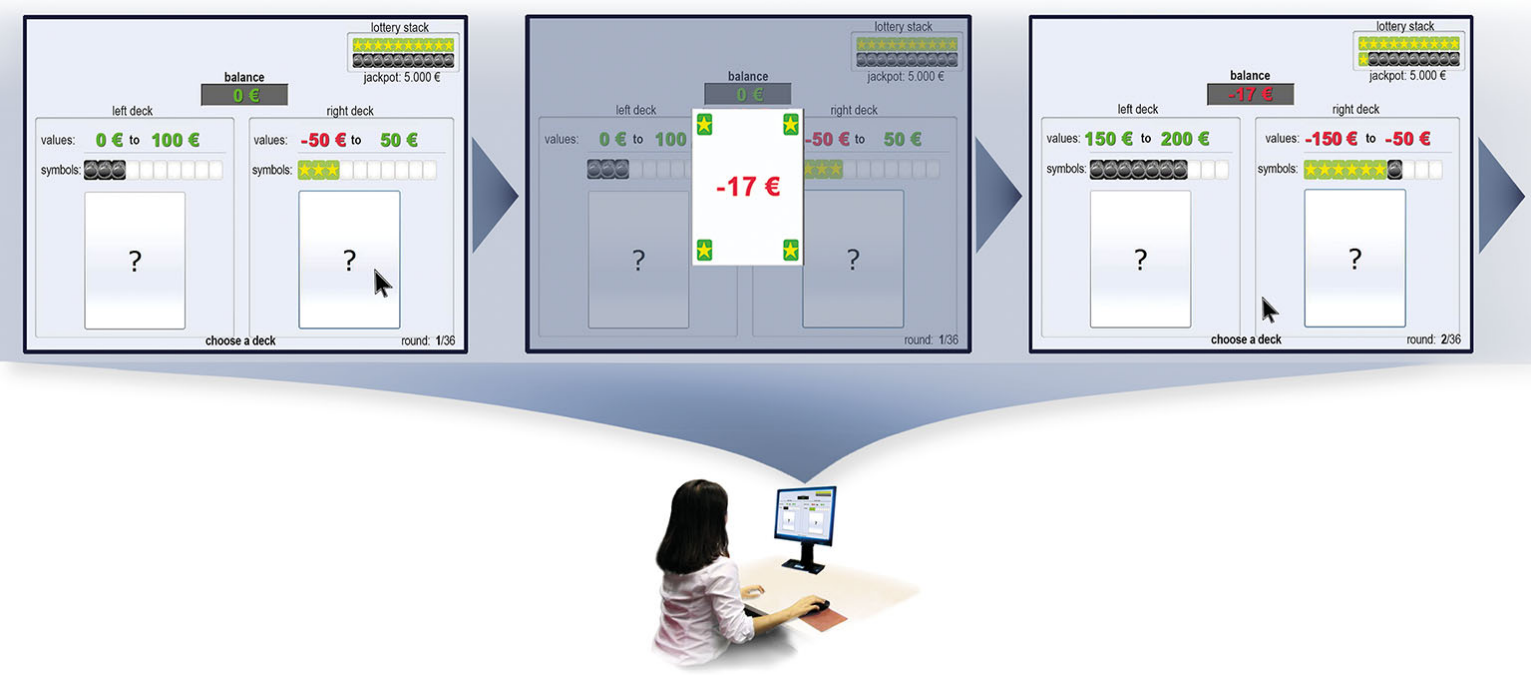
Illustration of a decision-making situation in the Cards and Lottery Task. The task starts with a balance of 0 EUR and a lottery stack of 10 stars versus 10 bombs. The left deck contains 10 cards with immediate gains ranging from 0 to 100 EUR, of which three additionally have bomb symbols and seven are neutral. The right deck contains 10 cards with values ranging from −50 EUR to 50 EUR, of which three additionally have star symbols and seven are neutral. In this example, the participant draws a card from the right deck. Feedback is immediately given about the card randomly drawn from the right deck, in this case a card with an immediate loss of −17 EUR and a star symbol. Participant’s balance and lottery stack are updated according to the card drawn previously. The next trial starts with altered win/loss margin and star/bomb symbol frequencies displayed above both decks.

The Cards and Lottery Task [CLT, (39)] is a computerized neuropsychological task assessing decision making under risk conditions. Participants were instructed to win as much virtual money as possible by choosing cards from two possible decks with conflicting short-term (i.e., win or loss of virtual money immediately added to or subtracted from the participant’s balance) and long-term consequences (i.e., chance to win or lose an additional jackpot in the subsequent lottery at the very end of the task). Each deck consisted of 10 cards displaying two characteristics: an amount of virtual money in EUR that was instantly added to or subtracted from the current balance after each draw (i.e., short-term) and a star or bomb symbol that influenced the probability of winning or losing the subsequent lottery (i.e., long-term; see **Figure 1** for an illustration and example). In the event of winning the lottery at the end, a further 5,000 EUR were added to the participants’ cumulated short-term balance, while 5,000 EUR were withdrawn from the balance if the lottery was lost. There were also neutral cards with no symbol and no effect on the outcome of the lottery. In each of the 36 trials, participants had to choose one card from either the left or right deck by mouse click, which was then randomly selected out of the 10 cards of the deck. The left deck included relatively high immediate gains and no losses of virtual money, however, was disadvantageous in the long-term (i.e., many bomb cards increasing the chance to lose the lottery jackpot). Contrary, the right deck included relatively low short-term gains or even loses, but was advantageous in the long term (i.e., many star cards increasing the probability to win the lottery jackpot). The win/loss margin and star/bomb-symbol frequencies in both decks altered in each trial and were displayed to participants as a decision basis. In addition, participant’s current balance of virtual money, the ratio of collected star and bomb symbols (i.e., lottery stack with 20 slots in total, starting at a portion of 10 stars *versus* 10 bombs, a drawn star replaces a bomb and *vice versa*), and the current trial number were constantly displayed on the screen and updated after each trial. Accordingly, participants had to integrate conflicting short- and long-term consequences of different options, as well as the current status of their short-term (i.e., balance of virtual money) and long-term (i.e., lottery stack) accounts during the decision-making process. The CLT was played in full feedback mode, meaning that after each decision, feedback in terms of a short visualization of the actually drawn card including its value and potential symbol was displayed in the center of the screen (see **Figure 1** for an example) and participant’s scores were immediately updated according to the properties of the drawn card. The visual feedback was accompanied by an auditory feedback indicating an immediate gain (positive sound) or loss (negative sound).

Risk-taking behavior and reward sensitivity were determined by the Number of Advantageous Decisions (NAD) score, indicating whether decision making is driven by immediate reward irrespective of negative outcomes in the long term or delay of immediate gratification (or acceptance of short-term punishment) in favor of greater, positive long-term outcomes. Although the highest overall outcome (i.e., final balance of gained virtual money) is only reached with winning the lottery jackpot at the end of the task, the NAD score is calculated in a way that exclusively choosing the right “long-term” deck is not the most advantageous strategy (e.g., choosing the long-term deck is not an advantageous decision if the lottery stack is already full of stars, i.e., 100% chance of winning the lottery is reached, and drawing another star card does not bring any additional benefit). Thus, the NAD score also takes the actual status of participant’s short-term and long-term accounts in each trial into consideration and thus represents objectively advantageous decisions in terms of choices for the option that offers the higher expected value in the respective situation [for a more detailed description, see (39)]. The NAD score ranges from 0 to 36, with lower scores indicating higher risk-taking behavior and lower delay of gratification.

### Measures for Validation

#### Convergent Validity

The *Delay Discounting Task* [DDT, (19)] determines reward sensitivity as aspect of impulsive decision making by assessing the individual tendency to discount the subjective value of a reward with increasing time that it takes to be released. Participants had to make a series of decisions, in which they could choose between either a fixed amount of money (10 EUR) with varying time delays (0, 2, 30, 180, and 365 days) or a varying amount of money (chosen randomly between 0 and 10 EUR in 0.5 increments) with no time delay. The task ends when an indifference point for each delay was found or the maximum of 30 trials for each delay was reached. Delay discounting was determined by calculating the Area Under the Curve [AUC, range: 0–1, (46)] based on the individual indifference points found for each delay, with lower values indicating higher discounting of delayed rewards (i.e., preference for short-term but lower amounts of money *vs.* long-term but higher amounts of money). In the present study, a computerized version of the DDT was applied, run by the test software Millisecond (Inquisit, Released 2015 for Windows, Version 4, Millisecond Software: San Francisco, CA, USA).

The short version of the *Barratt Impulsiveness Scale* [BIS-15, (47, 48)] was administered to assess self-reported impulsivity on three factors: non-planning, motor, and attentional impulsivity. Total sum scores (range: 15–60; Cronbach’s α in this study’s sample = .80) were calculated, with higher values indicating higher impulsivity.

The *Behavioral Inhibition System* (BIS) and *Behavioral Activation System* (BAS) scales (49, 50) were administered to assess reactive temperament as one underlying aspect of impulsive behavior. The BIS scale (range: 7–28; α = .81) measures individual dispositional differences in punishment sensitivity, whereas the BAS scale (range: 13–52; α = .80) assesses dispositional differences in reward sensitivity. Total sum scores were calculated for each scale, with higher values indicating higher sensitivity for punishment and reward, respectively.

The total sum score (range: 19–133; α = .73) of the *Effortful Control subscale of the Adult Temperament Questionnaire-Short Form* [ATQ-EC, (51, 52)] was calculated as a measure of self-control (i.e., individual’s capacity to overcome reactive approach tendencies, e.g., punishment or reward sensitivity, in order to act purposefully in the long term). Higher values indicate higher effortful control/self-control (i.e., lower impulsivity).

#### Discriminant Validity

The *Attention-Deficit/Hyperactivity Disorder Self-Rating Scale* [ADHD-SR, (53)] was used to assess self-reported attention-deficit/hyperactivity disorder (ADHD) symptoms according to the criteria “inattention,” “hyperactivity,” and “impulsivity” (α = .76) of the Diagnostic and Statistical Manual of Mental Disorders [DSM-5, (54)]. Participants were evaluated as probable cases of adult ADHD if they had at least six positive items (score > 0) from the items 1–9 (“inattention”) as well as at least six positive items from the items 10–18 [“hyperactivity,” “impulsivity,” (53)].

The binge-eating disorder module of the clinical interview *Eating Disorder Examination* [EDE, (55, 56)] was applied to assess binge eating (BE), defined as eating episodes, in which individuals experience a loss of control over eating, irrespective of consuming an objectively (objective BE episode) or subjectively (subjective BE episode) large amount of food in these situations (54). Participants were classified as having BE if they reported at least one objective and/or subjective BE episode over the past 3 months.

#### Clinical Validity

The *Patient Health Questionnaire Depression Scale* [(PHQ-9, (57, 58)] was used to screen for depression according to the nine diagnostic criteria of the DSM-5 (54). The total sum score (range: 0–27; α = .85) was calculated, with higher values indicating higher levels of depression.

The *Difficulties in Emotion Regulation Scale* [DERS, (59, 60)] was used to assess deficits in recognition and regulation of emotions. The total sum score (range: 36–180; α = .94) was calculated, with higher values indicating higher levels of emotion dysregulation.

Participants’ eating disorder psychopathology was evaluated *via* the *Eating Disorder Examination-Questionnaire* [EDE-Q, (61, 62)], assessing eating disorder attitudes and behaviors on the four subscales “restraint,” “eating concern,” “weight concern,” and “shape concern.” The EDE-Q global score (range: 0–6; α = .90) was calculated, with higher values indicating greater eating disorder psychopathology.

### Procedure

Experimental sessions were conducted individually and followed a standardized procedure. After obtaining written informed consent, the participant’s weight and height were measured objectively. Afterwards, visual attention towards food cues was determined using an eye-tracking and reaction-time paradigm (Schäfer et al., in revision), which were not part of the current analysis. Subsequently, the CLT and the DDT were conducted, followed by evaluating participant’s binge-eating episodes *via* EDE. Altogether, the experimental session took approximately 90 to 120 min. Self-report questionnaires were filled in at home beforehand. Of the total sample, 31 participants without intensive weight-loss treatment and 32 participants after obesity surgery were re-tested at a 1-year follow-up visit using the same procedure.

### Statistical Analysis

Statistical analyses were conducted using IBM SPSS Statistics version 23 (IBM Corp. Released 2015. IBM SPSS Statistics for Windows, Version 23.0. Armonk, NY: IBM Corp). Normal distribution and homogeneity of variances were confirmed before testing hypotheses. For all analysis, statistical significance was set at a two-tailed α level of .05.

To evaluate CLT performance (i.e., the NAD score) depending on sociodemography, independent sample *t* tests were conducted in dichotomous sociodemographic groups (women *vs.* men; age ≤ *vs.* > 45 years; education ≤ *vs.* > 10 school years). For convergent validity, Pearson correlations were calculated to determine associations between CLT and other measures of impulsivity (DDT, BIS-15, BIS/BAS, ATQ-EC). Regarding the CLT’s discriminant validity, mean NAD scores of the CLT were compared between clinical groups (ADHD symptoms *vs.* no ADHD symptoms; BE episodes *vs.* no BE episodes) as well as between the present sample and the population-based sample by Müller et al. (39) using independent sample *t* tests. If significant sociodemographic effects of the NAD score were detected, analyses on discriminant validity were controlled for these covariates using analyses of covariance, but only reported if changing the results. For clinical validity, Pearson correlations between CLT and measures of general psychopathology (PHQ-9, DERS), eating disorder psychopathology (EDE-Q), and BMI were conducted. Regarding test-retest reliability, Pearson correlations between two CLT performances measured one year apart in 31 participants without surgical or behavioral weight-loss treatment were run. A dependent sample *t* test was used to identify differences in CLT performance assessed pre- and 1-year post-surgery in 32 participants with surgical weight-loss treatment to evaluate the CLT’s sensitivity to change.

For estimating effect sizes, Pearson’s *r* for correlations (*r* = .10 small, .30 medium, .50 large) and Cohen’s *d* for *t* tests (*d* = .20 small, .50 medium, .80 large) were calculated and interpreted according to Cohen (63).

## Results

Boxplots of analyzed group differences (**Figure S1**), scatterplots of reported correlations (**Figure S2**), and bivariate correlations between all measures (**Table S3**) are included in the **supplementary material**.

### Sociodemographic Effects

Against expectation, CLT performance did not differ significantly between sociodemographic groups related to sex and age (*ps* > .05, small effects), as displayed in **Table 2**. As expected, participants with low *versus* high education exhibited a lower mean NAD score (*p* < .05, large effect).

**Table 2:** Cards and Lottery Task: The number of advantageous decisions (NAD score) by sociodemographic group.

	Group	*n*	CLT NAD Score	*t*	*df*	*p*	*d*
			*M (SD)*				
Sex	female	52	17.3 (6.7)	1.660	1, 76	.101	.40
	male	26	19.9 (6.2)				
Age (years)	24–45	46	18.5 (6.5)	0.581	1, 76	.563	.14
	46–69	32	17.6 (6.7)				
Education (school years)	≤10 >10	60 18	17.0 (6.3) 21.9 (6.1)	-2.902	1, 76	**.005**	.78

Significant p values are in boldface. CLT, Cards and Lottery Task; NAD, Number of Advantageous Decisions (0–36, lower scores indicate higher impulsivity).

### Convergent Validity

In line with hypotheses, a higher number of advantageous decisions in the CLT (NAD score) was associated with lower delay discounting (i.e., greater AUC in the DDT), *r* (71) = .33, *p* = .005, greater self-reported effortful control (ATQ-EC), *r*(76) = .23, *p* = .039, lower self-reported non-planning, motor, and attentional impulsivity (BIS-15), *r*(76) = −.36, *p* = .001, and lower punishment sensitivity (BIS), *r*(74) = −.29, *p* = .010. Against expectations, no significant association was found between the NAD score of the CLT and self-reported reward sensitivity (BAS), *r*(73) = .07, *p* = .576.

### Discriminant Validity

As shown in **Table 3**, participants with ADHD symptoms showed significantly lower mean NAD scores on the CLT than those without ADHD symptoms (*p* < .05, medium effect), as expected. After controlling for education, the effect was marginally significant (*p* = .057). No significant mean NAD score differences were found between participants reporting BE episodes and those without BE episodes (*p* > .05, small effect). Exploratory analyses on those with (*n* = 9) *versus* without (*n* = 69) full-syndrome BED revealed similar results (*p* = .335, small effect).

**Table 3:** Discriminant validity of the Cards and Lottery Task regarding different clinical groups.

	Group	*n*	CLT NAD Score	*t*	*df*	*p*	*d*
			*M (SD)*				
ADHD	ADHD symptoms	17	15.3 (5.6)	−2.038	1, 76	**.045**	.56
	no ADHD symptoms	61	18.9 (6.6)				
Binge Eating	BE episodes no BE episodes	29 49	16.8 (6.7) 18.9 (6.5)	−1.335	1, 76	.186	.31
Weight group	severe obesity^a^ healthy controls^b^	78 70	18.1 (6.6) 20.4 (5.2)	−2.356	1, 146	**.020**	.39

Significant p values are in boldface. CLT, Cards and Lottery Task; NAD, Number of Advantageous Decisions (0–36, lower scores indicate higher impulsivity); ADHD, attention-deficit/hyperactivity disorder; BE, binge eating.

^a^present sample (mean BMI = 48.1 ± 8.3 kg/m^2^), ^b^population-based sample by Müller et al. (39).

As hypothesized, the mean NAD score of the CLT differed significantly between individuals with severe obesity and the 70 healthy individuals who had performed the same CLT version in the study by Müller et al. [(39), *p* = .020, small effect], indicating more disadvantageous decision making in individuals with severe obesity versus healthy controls.

### Clinical Validity

As expected, the NAD score of the CLT was negatively correlated with depression (PHQ-D), *r*(76) = −.28, *p* = .012, and difficulties in emotion regulation (DERS), *r*(73) = −.28, *p* = .017, indicating that higher experimentally assessed impulsivity was significantly associated with higher levels of self-reported depression and emotion dysregulation. Against hypotheses, no significant correlations were found between participants’ performance in the CLT and self-reported eating disorder psychopathology (EDE-Q), *r*(75) = −.09, *p* = .459, and BMI, *r*(76) = .04, *p* = .743.

### Test-Retest Reliability

In the subsample of *n* = 31 participants without intensive weight-loss treatment, the test-retest reliability measured over one year (mean time interval = 12.5 ± 0.9 months, range: 11–14 months) was moderate with *r* = .40 (*p* = .026).

### Sensitivity to Change

In the subsample of *n* = 32 participants with obesity surgery, pre-surgery CLT performance (mean NAD = 19.0 ± 7.0) did significantly differ from post-surgery CLT performance (mean NAD = 15.6 ± 7.3; *t*(31) = 2.308, *p* = .028, Cohen’s *d* = .45) assessed at 1-year follow-up (mean time interval = 12.8 ± 0.7 months, range: 11–14 months), indicating an increase in risky decision making from pre- to 1-year post-surgery.

## Discussion

This study provided first psychometric evidence of a newly developed, complex measure on decision making in a clinical sample of adults with severe obesity, demonstrating highly favorable psychometric properties of the Cards and Lottery Task [CLT, (39)]. Specifically, the CLT’s convergent, discriminant, and clinical validity was shown using other well-established measures on delay discounting, effortful control, reward and punishment sensitivity, and depression. Over a period of one year, CLT performance showed moderate stability in those with constant body weight, but was sensitive to change in those who underwent obesity surgery and experienced substantial weight loss.

### Sociodemographic Effects

The present study did not replicate findings by Müller et al. (39) that sex and age have an effect on CLT performance, but goes in line with recent research consistently demonstrating a lack of effects of age and sex on decision making in individuals across the BMI range (17, 37, 41). A more important sociodemographic aspect in the research of decision-making abilities than age and sex is education (64). As expected and in line with recent evidence [e.g., (42, 65)], individuals with higher educational level outperformed those with lower education. A higher education is likely related to higher income which can be supported by the present data, where 56% of those with higher education had a net income ≥ 2,000 EUR (median value) compared to 31% of the low education group (χ²(1, *N* = 77) = 3.739, *p* = .051, *d* = .45; data not shown). The task of the CLT to maximize virtual money based on long-term decisions may thus be more familiar and realistic to individuals with higher education and financial security. It is thus imperative to consider participants’ level of education and income as important control variables in decision-making research.

### Convergent Validity

As expected, the number of advantageous decisions in the CLT was positively associated with the ability to wait for larger delayed rewards instead of preferring smaller immediate rewards during the classical DDT. Although the DDT is a measure of decision making based on explicit information, it differs from the CLT in central aspects including the lack of simultaneous short- and long-term consequences of each decision. Thus, the significant, but moderate association (*r* = .33) between both measures may depict the overlapping, yet distinct facets of decision making in individuals with severe obesity. Interestingly, the CLT (in the respective full feedback version) and DDT did not significantly correlate in a sample of healthy adults (39), suggesting that reward sensitivity is more relevant in decision-making processes in clinical samples with well-known deficient executive functioning than in healthy samples. Relatedly, the hypothesized significant association between advantageous decisions in the CLT and lower self-reported impulsivity and greater effortful control, respectively, underscored the CLT’s convergent validity in the present sample. In analogy to the DDT, these correlations were found only in the clinical, but not in the healthy validation sample by Müller et al. (39). In the latter sample, the mentioned correlation with self-reported impulsivity occurred only in case the CLT was performed in a modified version in which immediate feedback about long-term effects of the decision was hidden making short-term outcomes more salient. Against expectation, the BAS score was not associated with CLT performance. It might be hypothesized that the self-reported BAS score, representing the general tendency to act impulsively with high reward sensitivity and approach motivation, does not tap into the temporal component of conflicting short- and long-term prospects as the CLT does (66).

### Discriminant Validity

An important goal in the development of neuropsychological tasks is the ability to distinguish between different groups of individuals, mostly clinical and non-clinical samples. In fact, the present study showed that the CLT differentiated between individuals with severe obesity with and without symptoms of ADHD. In the CLT, adults with symptoms of ADHD, a neurodevelopmental disorder associated with deficient executive control (67) and decision-making deficits which are of comparable magnitude as attention deficits (68), made significantly more disadvantageous decisions than those without ADHD symptoms. Compared to the only available validation study of the CLT in healthy participants from the population (39), individuals with severe obesity made significantly more disadvantageous decisions, which is in line with *a priori* expectations and evidence on impaired decision making in individuals with obesity *versus* normal-weight controls (17). Contrary to expectations, CLT performance did not differ between individuals with *versus* without binge-eating episodes, which might be related to the subthreshold definition and frequency of objective or subjective binge-eating episodes in the present study. Indeed, in the present sample binge-eating prevalence was very low coupled with high variability. Future studies on the CLT are highly recommended to investigate full syndrome binge-eating disorder including regularly occurring objectively large binge-eating episodes according to the DSM-5 (54).

### Clinical Validity

As expected and consistent with the diagnostic criteria of a major depressive disorder including a “diminished ability to think or concentrate, or indecisiveness” (55), individuals reporting higher compared to lower levels of depression showed more disadvantageous decision making. In fact, previous studies using the IGT demonstrated deficient decision making in individuals with depressive disorders compared to controls [e.g., (69, 70)]. At the same time, depressive disorders go along with behavioral hypersensitivity to punishment [e.g., (71–73)] and behavioral and biological hyposensitivity for positive reinforcements (74), which may explain the preference for avoiding the deck with immediate punishment (but greater, positive long-term outcomes). Relatedly, self-reported emotion regulation difficulties covering, for example, difficulties engaging in goal-directed behavior and impulse control (59) were associated with deficient decision making in the present sample. This result goes in line with previous evidence demonstrating robust associations between greater impulsivity and emotion dysregulation in individuals with severe obesity (75). Contrasting expectations, lower CLT performance was not related to higher levels of eating disorder psychopathology in the present sample. In analogy to the lack of CLT differences in binge-eating status, this suggests that general decision-making abilities are not a function of domain-specific psychopathology such as eating disorder psychopathology. In this context, previous research did not provide consistent evidence on the association between weight and shape concern and measures of urgency (76).

### Test-Retest Reliability

Analyses on the task’s test-retest reliability revealed encouraging results, as CLT performance was moderately stable in the present sample. Of note, the test-retest interval was very long with more than 12 months on average. Comparable tasks assessing behavioral impulsivity showed higher test-retest reliability in previous studies, such as the DDT (*r* = .50–.89) and the Balloon Analogue Risk Task (*r* = .66–.79), but not the IGT [*r* = .27–.65, (44, 77–79)]. However, test-retest reliability in these studies was tested over much shorter time intervals ranging from 1 h to 3 weeks, with decreasing test-retest reliability over longer time intervals. Additionally, test-retest reliability of other neuropsychological measures of impulsivity was evaluated in non-clinical samples of individuals with younger age [≤ 35 years, (44, 77–79)] and higher educational level [e.g., undergraduate students, (77, 78)] compared to the present sample. These sociodemographic differences in combination with longer time intervals in the present study may result in greater sociodemographic heterogeneity in the current sample including a greater probability for changes in participants’ living conditions over time (e.g., job change, retirement) which potentially effected the temporal stability of CLT performance. Furthermore, learning effects (e.g., changing strategy after experiencing that only winning the lottery jackpot leads to the highest overall outcome in the CLT) and daily mood fluctuations (44) must always be taken into account when interpreting the test-retest reliability of neuropsychological tasks assessing impulsive decision making.

### Sensitivity to Change

The study revealed that CLT performance significantly changed after obesity surgery. Against hypotheses that decision making will improve after obesity surgery (25), however, CLT performance worsened from pre- to post-surgery. Participants made significantly more disadvantageous decisions than before surgery indicating that individuals experiencing substantial weight loss showed a higher tendency to prefer short-term over long-term rewarding options one year after obesity surgery. Notably, extant research on the effects of obesity surgery on psychopathology revealed substantial reductions in depressive and anxiety symptoms (80) as well as an increase in social and physical activity (81, 82). These liberating effects of obesity surgery may account for the increase in risk-taking behaviors with both positive and negative consequences, including leaving an unhealthy partnership, starting a new relationship (83), or post-surgery suicides (84). The favorable and detrimental effects of extreme weight loss on decision making should be subject of further investigation.

### Strengths and Limitations

A major strength of this study is the recruitment of a clinical sample with severe obesity representing a broad spectrum of age and education. Measures for validation were assessed multimodally using well-established neuropsychological tasks, clinical interviews, and self-report questionnaires. The longitudinal design allowed for testing the long-term stability of CLT performance in individuals with and without stable weight profile. Nevertheless, it should be taken into account that it was not controlled for symptoms of gambling disorders, which might be assumed to have an effect on CLT performance as the CLT depicts a (virtual) gambling task. CLT and DDT were presented in a non-randomized order, which bears the risk of mutual influence on test performance. However, the risk was considered to be minimal as the applied tasks were not reaction-time based, making distortions, e.g., due to fatigue, unlikely, and both paradigms tapped into different aspects of decision making. The CLT was only compared with the DDT—a decision-making measure outside the learning context as well—but not with the IGT. A direct comparison with the IGT would clarify the hypothesized task differences, especially the impact of punishment sensitivity on decision-making performance. As only virtual money was played for in the CLT, the transferability to real decision making is limited. An additional monetary incentive in the form of an achievable real amount of money in future study designs (e.g., 0.1% of the final account balance achieved in the CLT) would bring the CLT even closer to everyday life. Finally, although statistical power was appropriate for correlation analyses, tests for group differences were underpowered due to varying group sizes and should be carefully interpreted.

### Conclusion

The CLT was found to be a valid and reliable computerized task for the brief assessment of flexible decision making under explicit risk in a clinical sample of individuals with severe obesity. With more facets of decision making being captured than in the IGT and DDT, relatively short task duration (about 20 min) and pleasing design, it is a recommended task for research on decision making in population-based and clinical samples, specifically samples with severe obesity. Nevertheless, replication studies across the BMI range are needed to underpin the present results and to directly evaluate weight-specific differences in CLT performance. For further evaluation of a potential overlap with decision making under ambiguous risk, studies should validate the CLT against the widely-used IGT. Finally, it will be highly valuable to evaluate the CLT’s predictive value for real-life health behaviors in the short and long term as well as pathological risk-taking behaviors, such as drug and alcohol abuse, to assess its clinical utility more deeply.

## Data Availability Statement

The datasets generated for this study are available on request to the corresponding authors.

## Ethics Statement

This study involving human participants was reviewed and approved by the ethics committee of the Medical Faculty, Leipzig University, Germany. All participants provided their written informed consent to participate in this study.

## Author Contributions

LS, RS, and AH contributed the conception and design of the study. SM provided the CLT and respective scoring algorithms. LS performed the study and organized the database. LS and RS performed the statistical analysis and contributed to the interpretation of data. LS and RS wrote the original draft of the manuscript. All authors contributed to the article and approved the submitted version. AH acquired funding for this research.

## Funding

This work was supported by the Federal Ministry of Education and Research (BMBF), Germany, FKZ: 01EO1501, and we acknowledge support from the German Research Foundation (DFG) and University of Leipzig within the program of Open Access Publishing.

## Conflict of Interest

The authors declare that the research was conducted in the absence of any commercial or financial relationships that could be construed as a potential conflict of interest.
