# Supplementary material for: The Cards and Lottery Task: Validation of a New Paradigm Assessing Decision Making Under Risk in Individuals With Severe Obesity
Source: Front Psychiatry. 2020 Jul 16;11:690. doi: 10.3389/fpsyt.2020.00690 (PMC7378783; doi:10.3389/fpsyt.2020.00690)
Supplement: Supplementary file 1 [file DataSheet_1.docx]

Supplementary Material

# Supplementary Figures


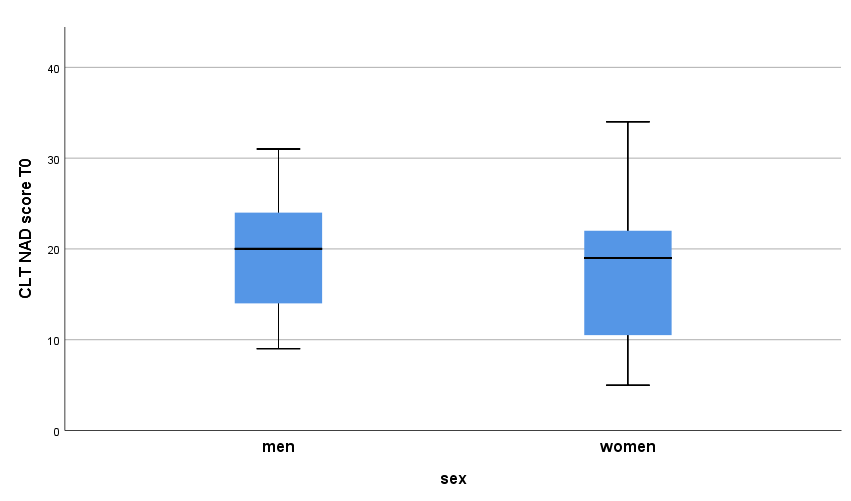

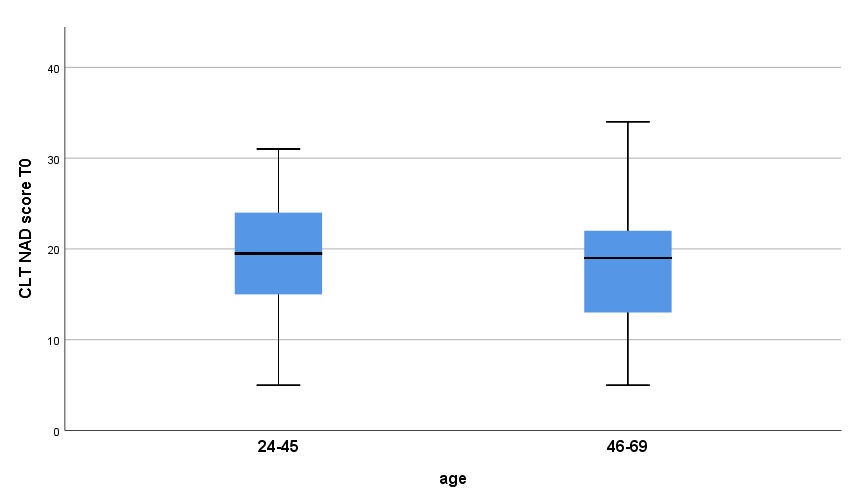
1a) 1b)


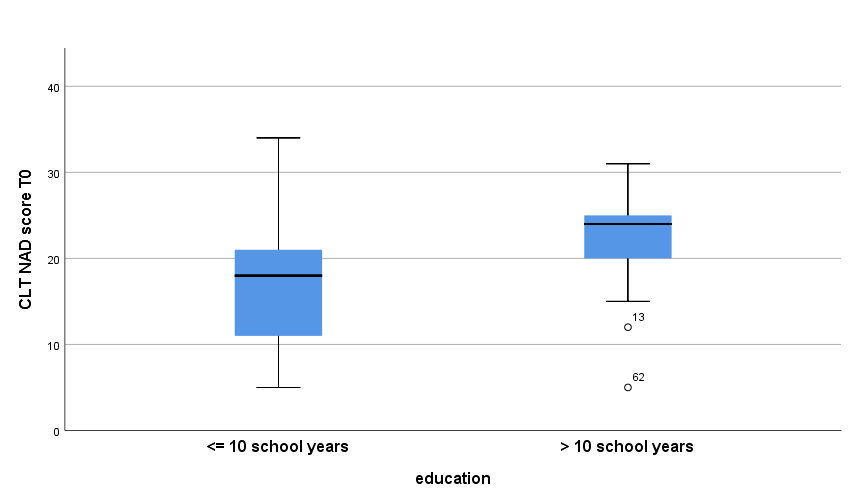

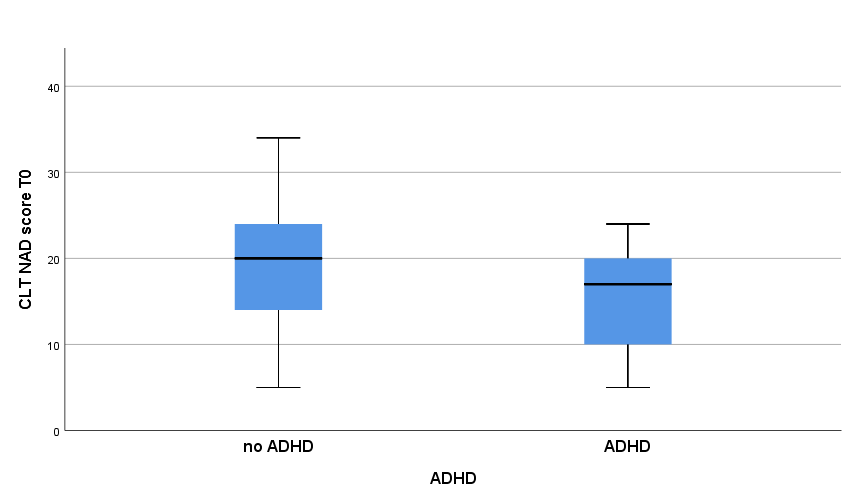
1c) 1d)


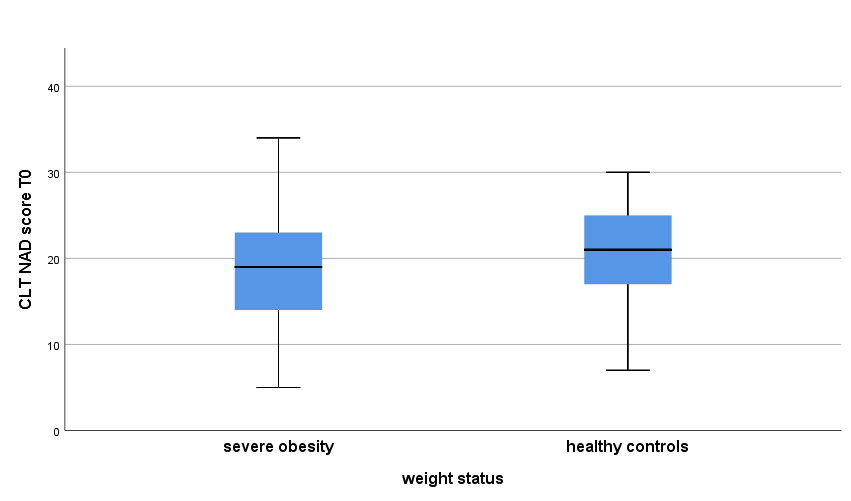

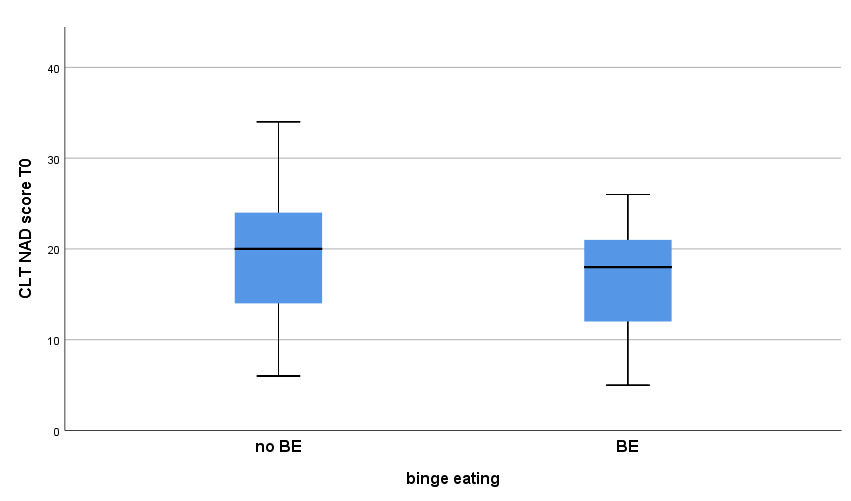
1e) 1f)

1g)


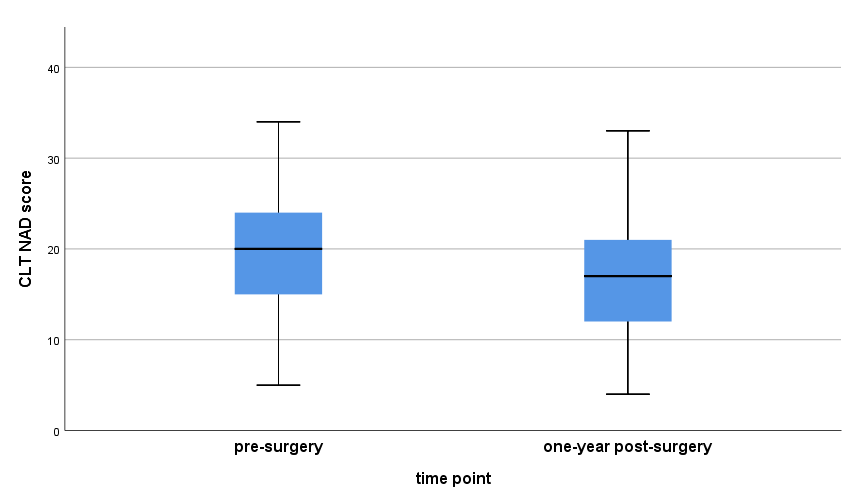


**Supplementary Figures 1a-g.** Differences in CLT performance depending on sociodemography (1a-1c), clinical group assignment (1d-1f), and assessment point in patients with obesity surgery (sensitivity to change, 1g). ADHD = Attention-Deficit/Hyperactivity Disorder; BE = Binge Eating; CLT = Cards and Lottery Task; NAD = Number of Advantageous Decisions.


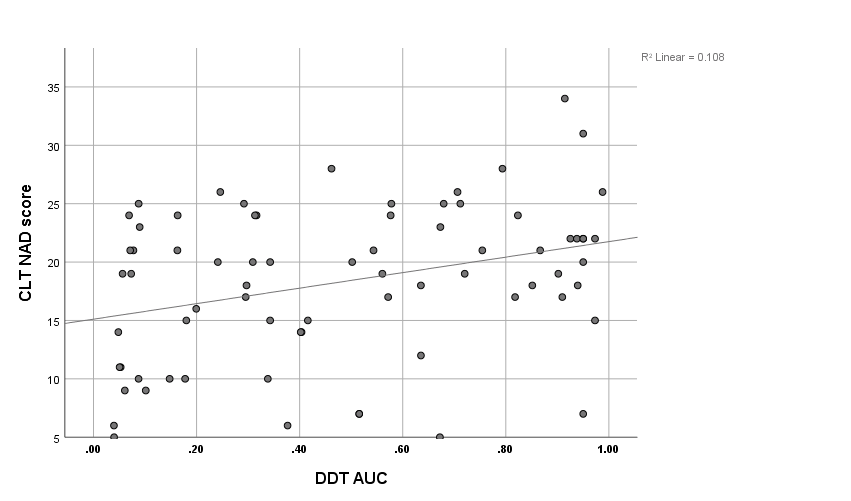
2a)


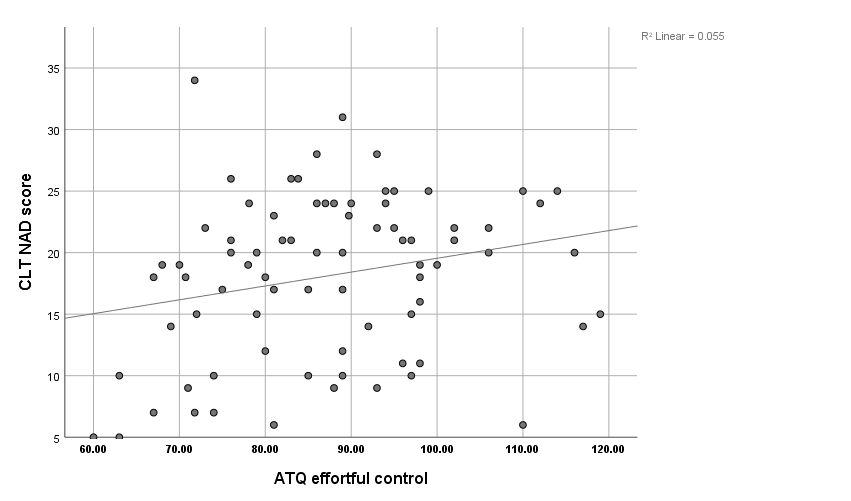


2b)


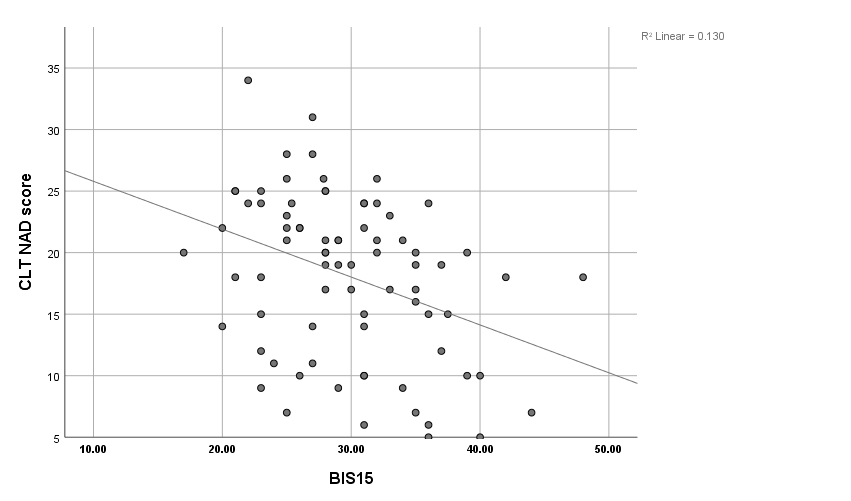


2c)


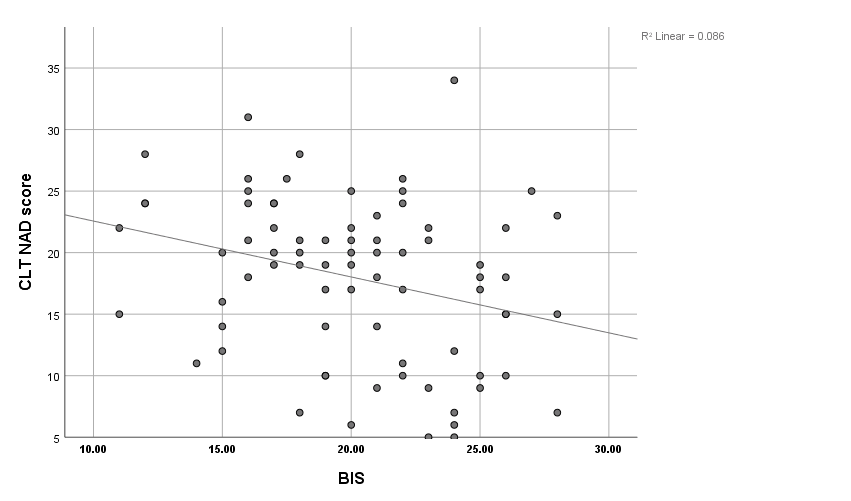
2d)


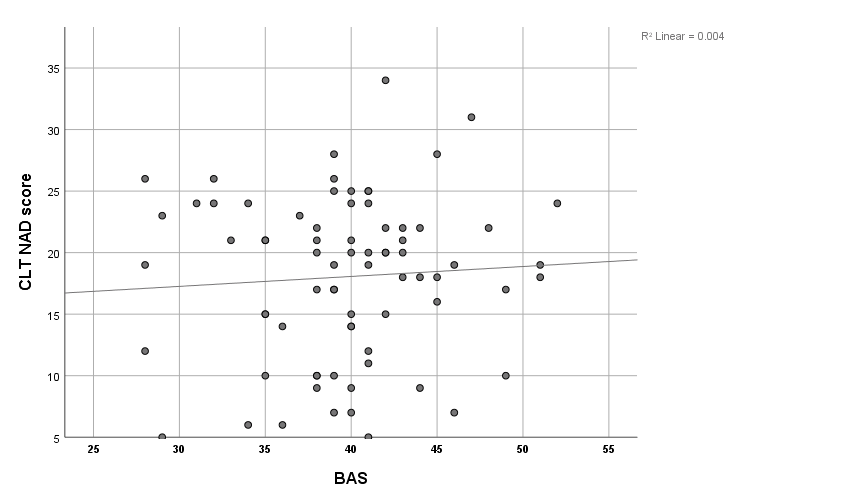


2e)


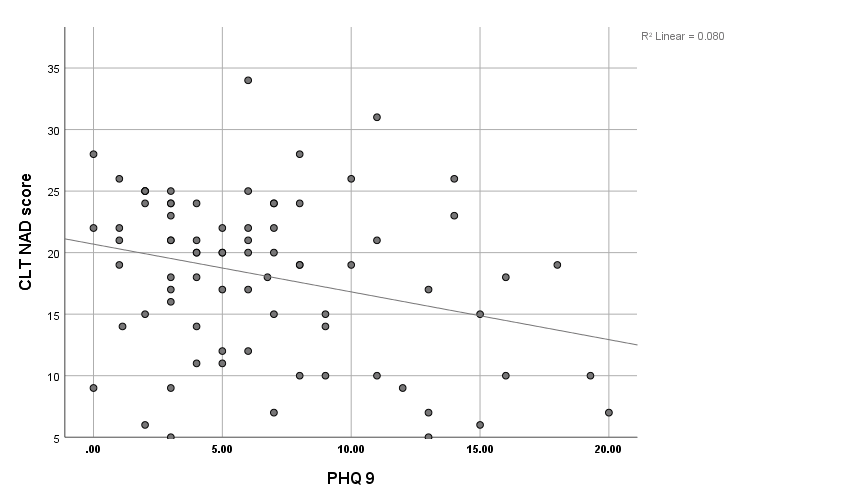


2f)


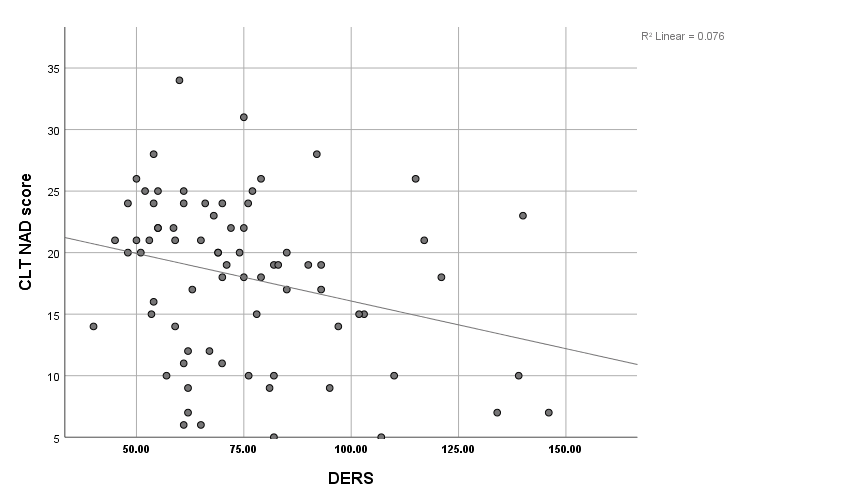
2g)


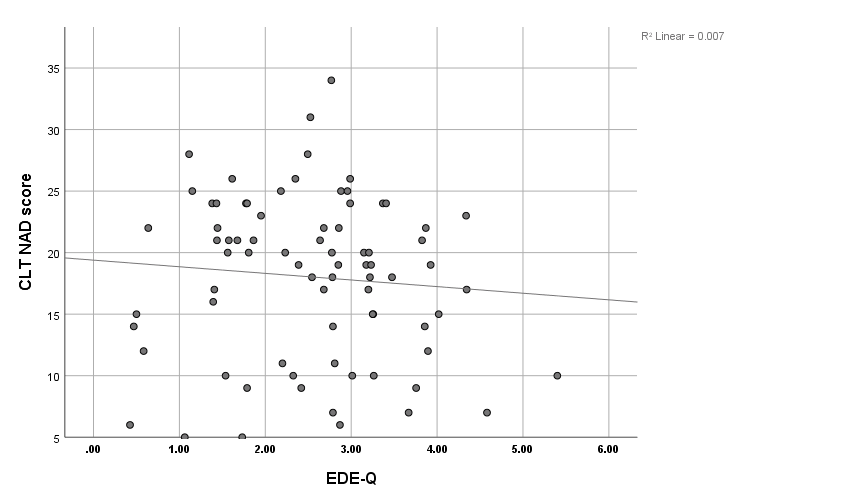


2h)


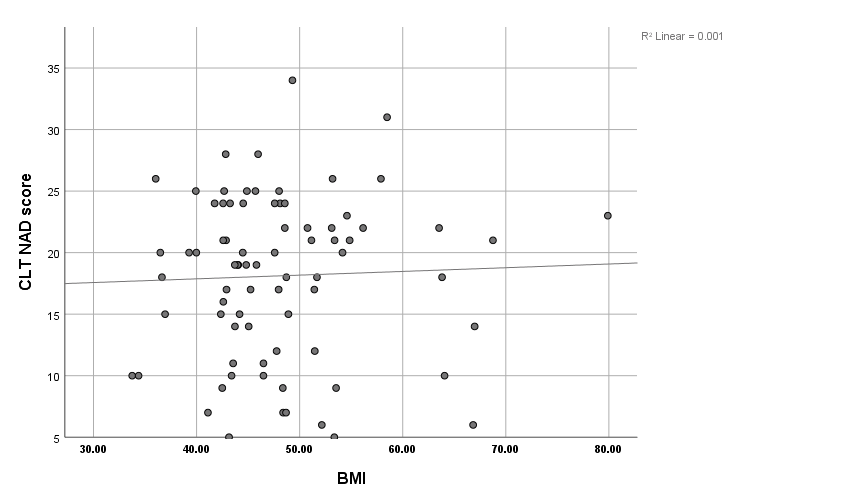


2i)


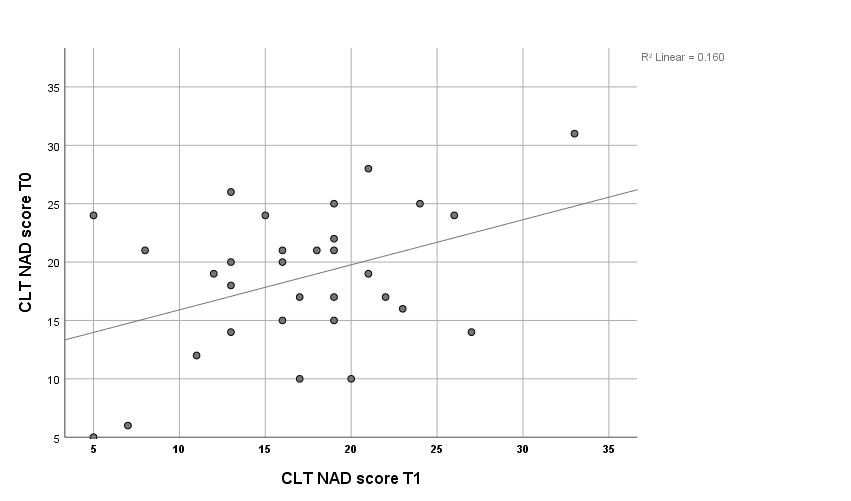
2j)

**Supplementary Figures 2a-j.** Scatterplots of Pearson correlations between baseline CLT performance and other measures of impulsivity (2a-2e) and clinical variables (2f-2i), and test-retest-correlation at 1-year follow-up (2j). ATQ = Adult Temperament Questionnaire; BAS = Behavioral Activation System; BIS = Behavioral Inhibition System; BIS-15 = Barratt Impulsiveness Scale – short version; BMI = Body Mass Index; CLT = Cards and Lottery Task; DERS = Difficulties in Emotion Regulation Scale; DDT = Delay Discounting Task; EDE-Q = Eating Disorder Examination-Questionnaire; NAD = Number of Advantageous Decisions; PHQ-9 = Patient Health Questionnaire Depression Scale.

# Supplementary Table

Table S3*. Correlations between Number of Advantageous Decisions of the Cards and Lottery Task (CLT NAD), Delay Discounting Task (DDT), Barrett Impulsiveness Scale (BIS-15), Behavioral Inhibition System (BIS) and Behavioral Activation System (BAS), Effortful Control subscale of the Adult Temperament Questionnaire-Short Form (ATQ-EC), Attention-Deficit/Hyperactivity Disorder Self-Rating Scale (ADHD-SR), Patient Health Questionnaire Depression Scale (PHQ-9), Difficulties in Emotion Regulation Scale (DERS,) and Eating Disorder Examination-Questionnaire (EDE-Q).*

|  | **CLT NAD** | **DDT** | **BIS-15** | **BIS** | **BAS** | **ATQ-EC** | **ADHD-SR** | **PHQ-9** | **DERS** | **EDE-Q** |
| --- | --- | --- | --- | --- | --- | --- | --- | --- | --- | --- |
| **CLT NAD** | 1 | .33** | -.36** | -.29** | .07 | .23* | -.28* | -.28* | -.28* | -.09 |
| **DDT** |  | 1 | -.09 | -.03 | .23 | -.02 | -.02 | .01 | .06 | .05 |
| **BIS-15** |  |  | 1 | .10 | -.12 | -.36** | .45*** | .40*** | .41*** | .12 |
| **BIS** |  |  |  | 1 | -.05 | -.36** | .33** | .39*** | .58*** | .48*** |
| **BAS** |  |  |  |  | 1 | .01 | -.06 | -.27* | -.09 | .07 |
| **ATQ-EC** |  |  |  |  |  | 1 | -.41*** | -.25* | -.50*** | -.10 |
| **ADHD-SR** |  |  |  |  |  |  | 1 | .52*** | .46*** | .29** |
| **PHQ-9** |  |  |  |  |  |  |  | 1 | .65*** | .60*** |
| **DERS** |  |  |  |  |  |  |  |  | 1 | .50*** |
| **EDE-Q** |  |  |  |  |  |  |  |  |  | 1 |

* *p* < .05, ** *p* < .01, *** *p* < .001
